# Distribution and Genetic Diversity of Genes Involved in Quorum Sensing and Prodigiosin Biosynthesis in the Complete Genome Sequences of *Serratia marcescens*

**DOI:** 10.1093/gbe/evz046

**Published:** 2019-03-06

**Authors:** Ryohei Sakuraoka, Tomohiro Suzuki, Tomohiro Morohoshi

**Affiliations:** 1 Department of Innovation Systems Engineering, Graduate School of Engineering, Utsunomiya University, Japan; 2 Center for Bioscience Research and Education, Utsunomiya University, Japan; 3 Department of Material and Environmental Chemistry, Graduate School of Engineering, Utsunomiya University, Japan

**Keywords:** *Serratia marcescens*, quorum sensing, N-acylhomoserine lactone, prodigiosin, comparative genomics, complete genome

## Abstract

Quorum sensing is a cell density-dependent regulation of gene expression. *N*-acyl-l-homoserine lactone (AHL) is a major quorum-sensing signaling molecule in gram-negative bacteria and synthesized by the LuxI family protein. The genus *Serratia* is known as a producer of the red pigment, prodigiosin, whose biosynthesis is dependent on the *pig* gene cluster. Some *Serratia* strains regulate prodigiosin production via AHL-mediated quorum sensing, whereas there is red-pigmented *Serratia* strains without quorum-sensing system. In addition, nonpigmented *Serratia marcescens*, which does not produce prodigiosin, has also been isolated from natural and clinical environments. In this study, we aim to reveal the distribution and genetic diversity of quorum-sensing genes and *pig* gene cluster in the complete genome sequences of *S. marcescens*. We previously demonstrated that *S. marcescens* AS-1 regulates the production of prodigiosin via AHL-mediated quorum sensing. We sequenced the genomes of AS-1 and compared with the complete genomes of AS-1 and the other 34 strains of *S. marcescens*. The *luxI* homolog was present on 25 complete genome sequences. The deduced amino acid sequences of the *luxI* homolog were divided into three phylogenetic classes. In contrast, the *pig* gene cluster was present in the genome of seven *S. marcescens* strains and only two strains, AS-1 and N4-5 contained both the *luxI* homolog and *pig* gene cluster in their genome. It is therefore assumed that prodigiosin production and its regulation by quorum sensing are not essential for the life cycle of *S. marcescens*.

## Introduction

*Serratia* is a genus of rod-shaped gram-negative bacteria and a member of the family *Enterobacteriaceae* (Van Houdt, Givskov, et al. 2007). Some strains belonging to the genus *Serratia* are capable of producing a red-pigmented secondary product, called prodigiosin (2-methyl-3-pentyl-6-methoxyprodiginine) (Williamson et al. 2006). Prodigiosin is reported to have antifungal, antibacterial, antiprotozoal/antimalarial, immunosuppressive, and anticancer activities; inducing the apoptosis of primary human cancer cells (Williamson et al. 2005). The biosynthesis of prodigiosin in the genus *Serratia* is dependent on the *pig* gene cluster consisting of *pigA-N* or *pigA-O* (Harris et al. 2004; Van Houdt, Givskov, et al. 2007). In contrast, nonpigmented *Serratiamarcescens*, which do not produce prodigiosin, have been isolated from natural and clinical environments (Carbonell et al. 2000). Both pigmented and nonpigmented *Serratia* strains are pathogenic for humans. However, there is concern that nonpigmented strains are more virulent due to cytotoxin production and antibiotic resistance (Roy et al. 2014).

Quorum sensing is a gene regulatory system that is stimulated in response to an increase in population density (Atkinson and Williams 2009). Some gram-negative *Proteobacteria* produce *N*-acyl-l-homoserine lactone (AHL), which is used as a signaling molecule involved in quorum sensing (Parsek and Greenberg 2000). The LuxI family protein encoded by *luxI* gene catalyzes the formation of AHL from *S*-adenosyl-l-methionine and acyl-acyl carrier proteins, or CoA-aryl/acyl moieties (Dong et al. 2017). AHL binds to the LuxR family protein encoded by *luxR* gene as an AHL receptor and controls the transcription of target genes (Parsek and Greenberg 2000). The prodigiosin biosynthetic pathway is controlled by a quorum-sensing system in some *Serratia* strains (Van Houdt, Givskov, et al. 2007). For instance, *Serratia* sp. ATCC 39006 produces *N*-butyryl-l-homoserine lactone (C4-HSL) and *N*-hexanoyl-l-homoserine lactone (C6-HSL). It regulates the production of prodigiosin, carbapenem, pectate lyase, and cellulase (Thomson et al. 2000). *Serratiamarcescens* SS-1 produces two major AHLs, *N*-(3-oxohexanoyl)-l-homoserine lactone (3-oxo-C6-HSL) and C6-HSL. It regulates the sliding motility and production of prodigiosin (Horng et al. 2002). In contrast, *S. marcescens* CH-1, which can produce prodigiosin, does not contain a LuxIR quorum-sensing system (Wei et al. 2006). The relationship between prodigiosin production and its regulation via quorum-sensing in *S. marcescens* therefore remains unclear. Previously, we demonstrated that *S. marcescens* AS-1 produces 3-oxo-C6-HSL and C6-HSL, and regulates the production of prodigiosin and swarming motility (Morohoshi et al. 2007). In this study, we report the complete genome sequence of AS-1 and the distribution of the *luxI* homolog and *pig* gene cluster in the reported complete genome sequences of *S. marcescens*.

## Materials and Methods

*Serratia* *marcescens* AS-1, which produces AHLs and prodigiosin, was isolated from a soil sample (Morohoshi et al. 2007). The strain AS-1 was cultured in Luria-Bertani broth for 18 h at 30 °C with shaking. Total genomic DNA was extracted from the overnight culture using a DNeasy Blood and Tissue Kit (Qiagen K.K., Tokyo, Japan) according to the manufacturer's protocol.

Genome sequencing of *S. marcescens* AS-1 was performed on the PacBio RSII platform (Pacific Biosciences, Menlo Park, CA) using libraries prepared with the SMRTbell Template Prep Kit 1.0 (Pacific Biosciences) by Macrogen Japan Corp. (Kyoto, Japan). The sequencing reads were assembled using Canu version 1.7 (Koren et al. 2017). Two circular contigs were subjected to the prediction and annotation of genes using the DFAST pipeline (Tanizawa et al. 2018). The coding sequences (CDSs) were predicted using Prodigal 2.6.3 (Hyatt et al. 2010). Genes coding for tRNA and rRNA were discovered using Aragorn 1.2.38 (Laslett and Canback 2004) and Barrnap 0.8 (https://github.com/tseemann/barrnap, last accessed February 7, 2019), respectively. The graphical map of the circular genome was generated using the CGView server (Grant and Stothard 2008).

The 34 complete genome sequences of *S. marcescens* were retrieved from the NCBI Genome website (https://www.ncbi.nlm.nih.gov/genome, last accessed February 7, 2019) as of October 15, 2018. The homology search was carried out using the in silico Molecular Cloning Genomics Edition (In Silico Biology Inc., Yokohama, Japan). The phylogenetic tree based on the LuxI sequences was constructed using the Neighbor-Joining method with the ClustalW of MEGA version 7.0 (Kumar et al. 2016). The phylogenetic tree based on the whole genome alignments was constructed using REALPHY 1.12 (Bertels et al. 2014).

## Results and Discussion

**Fig. 1.**
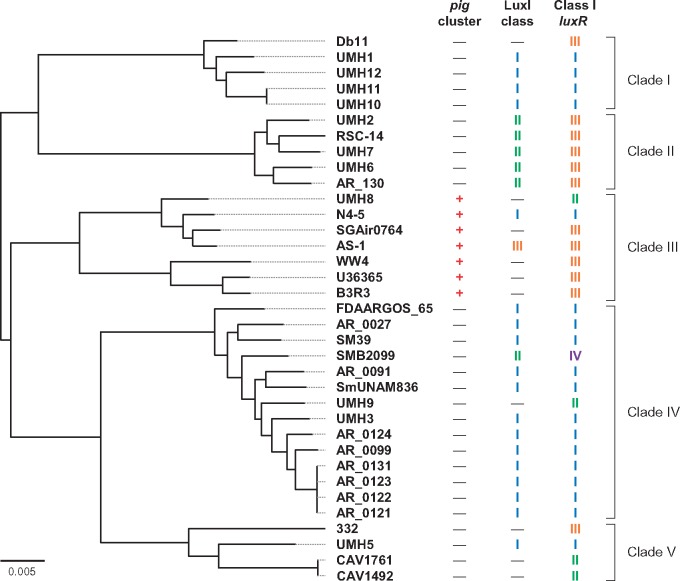
—Phylogenetic tree based on the complete sequences of chromosome of 35 strains of *Serratia marcescens*. The phylogenetic tree was constructed by REALPHY 1.12. The presence of *pig* gene cluster, classification of LuxI, gene arrangement around the class I *luxR* homolog, and phylogenetic clades were described on the right side of the tree.

The sequencing of the AS-1 genome using the PacBio RSII platform resulted in 97,080 reads, with an average read length of 15,150 bases. The total number of sequenced bases was 1,470,807,268, producing a representative sequencing depth of 284×, which sequenced ∼1.47 Gbp. Using the Canu assembler version 1.7, these reads were assembled into two large scaffolds, 5,097,044 and 130,881 bp. These linear scaffolds with overlapping ends were converted to closed circular DNA. Finally, the complete genomic information of the AS-1 is contained on a single chromosome of 5,071,908 bp with an average G + C content of 59.6%, and a single endogenous plasmid pSERAS01 of 104,121, with an average G + C content of 54.8% (supplementary fig. S1, Supplementary Material online). The chromosome contains 4,657 CDSs, 22 rRNA genes organized into seven rRNA operons, 93 tRNA genes, and one tmRNA. The plasmid pSERAS01 contains only 94 CDSs. To compare the genome sequences across different strains in *S. marcescens*, we obtained 34 complete genome sequences of *S. marcescens* deposited in the DDBJ/ENA/GenBank databases (supplementary table S1, Supplementary Material online). The phylogenetic tree based on the complete sequences of chromosome was split the 35 strains into five clades (fig. 1). Some strains of *S. marcescens* retain endogenous plasmids in their genome. The size of exogenous plasmids from these *S. marcescens* strains is different each other and range from 3 to 200 kbp. The plasmid pSERAS01 from AS-1 shows a partially high identity with an unnamed plasmid from SGAir0764 (data not shown).

**Fig. 2.**
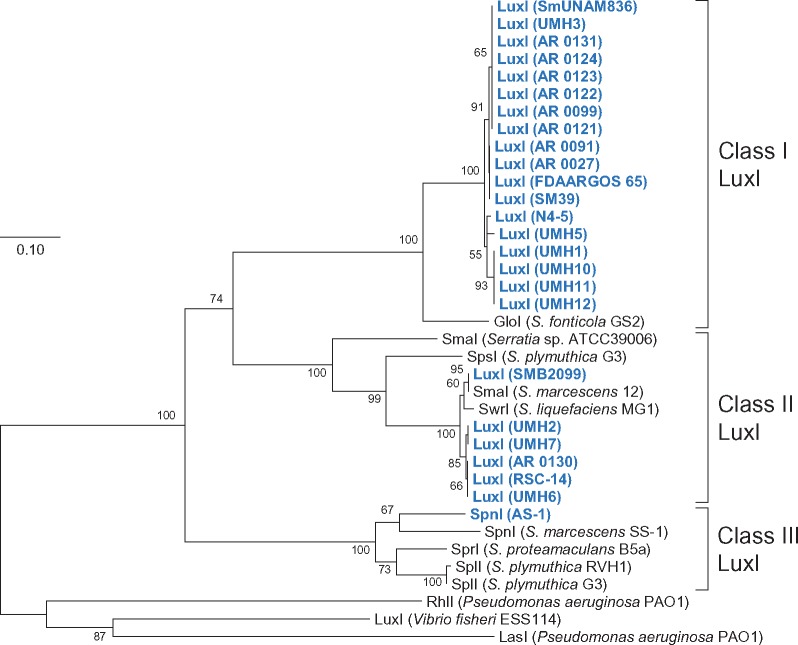
—Phylogenetic tree based on amino acid sequences of LuxI homologs from the genus *Serratia*. The phylogenetic tree was constructed using the Neighbor-Joining method with the ClustalW program of MEGA7. The percentage of replicate trees in which the associated taxa clustered together in the bootstrap test (1,000 replicates) is shown next to the branches. The scale bar represents 0.10 substitutions per amino acid position. LuxI from *Vibrio fischeri* ESS114 (UniProt accession no. P35328), RhlI (P54291), and LasI (P33883) from *Pseudomonas aeruginosa* PAO1 were used as outgroup. The LuxI homologs from the complete genome sequences are shown in blue.

We searched for the presence of the *luxI* homolog, in the complete genome of 35 strains of *S. marcescens*. The results of the BLAST search revealed that the 25 complete genomes contained the gene sequence of the *luxI* homolog (fig. 1). The deduced amino acid sequences of the LuxI homolog were classified with multiple sequence alignments. On the basis of the results of the phylogenetic analysis, these LuxI homologs were divided into three classes at an identity level of 95% (fig. 2). Class I was the most dominant class, containing 18 LuxI homologs. Class II was the next dominant class, containing 6 LuxI homologs. Class III contained only one LuxI homolog, which was obtained from the AS-1 genome.

**Fig. 3.**
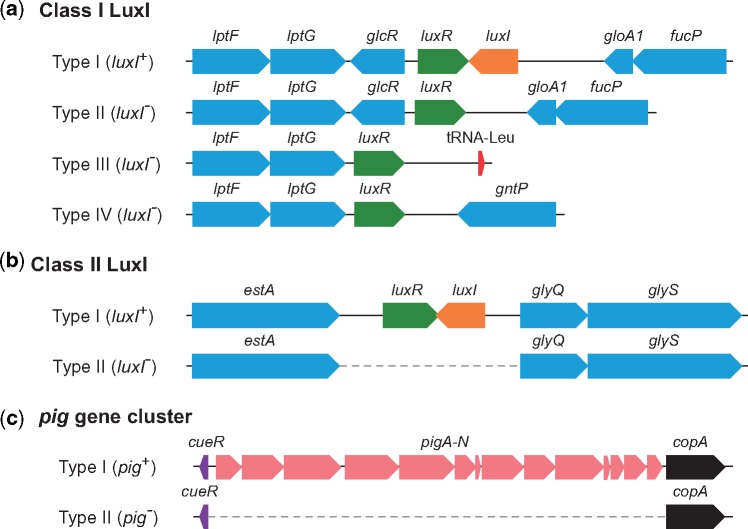
—The arrangement of the predicted ORFs around the *luxI*/*luxR* belonging to class I (*a*) and II (*b*), and *pig* gene cluster (*c*). Genes and their orientations are depicted with arrows using the following colors: orange, *luxI* homolog; green, *luxR* homolog; pink, *pig* gene cluster; purple, *cueR*; black, *copA*; blue, other genes. The dashed line represents a deleted region on the chromosome.

The class I LuxI showed a similarity with AHL synthase GloI from *Serratiafonticola* GS2 (DDBJ/ENA/GenBank accession no. KX257356). It has been reported that GS2 produces two AHLs, C6-HSL and *N*-octanoyl-l-homoserine lactone (C8-HSL; Jung et al. 2017). The class I *luxI* homolog and its associated *luxR* homolog were placed downstream of gene homologs involved in lipopolysaccharide export system permease (*lptFG*) and between gene homologs involved in DeoR family transcriptional regulator (*glcR*) and glyoxalase (*gloA1*) (fig. 3*a*). Interestingly, regardless of the presence or absence of the class I *luxI* homolog, the *luxR* homolog existed downstream of the *lptFG* homologs in the complete genome sequences of all strains. The gene arrangement around the *luxR* homolog was classified into four types (figs. 1 and 3*a*).

The class II LuxI showed similarities with SmaI from *S. marcescens* 12 (Coulthurst et al. 2006), SwrI from *S. liquefaciens* MG1 (Givskov et al. 1998), SmaI from *Serratia* sp. ATCC39006 (Thomson et al. 2000), and SpsI from *S. plymuthica* G3 (Liu et al. 2011). These reported LuxI homologs similarly catalyze the biosynthesis of C4-HSL and C6-HSL as major AHLs. The class II *luxI* homolog and its associated *luxR* homolog were seated between gene homologs involved in outer membrane lipase (*estA*) and glycine tRNA synthetase (*glyQ*), but completely disappeared between *estA* and *glyQ* in the genome of class II LuxI-negative strains (fig. 3*b*).

**Fig. 4.**
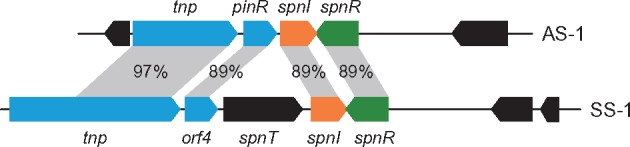
—Comparison of the *spnIR* gene and its upstream region from *Serratia marcescens* AS-1 and SS-1. Genes and their orientations are depicted with arrows using the following colors: orange, *spnI*; green, *spnR*; blue, *pinR* and transposase (*tnp*); black, other genes. The identity values of the corresponding gene products are represented between the gray area.

As for class III LuxI, we have already reported that SpnI from AS-1 produced two AHLs, C6-HSL and 3-oxo-C6-HSL (Morohoshi et al. 2007). SpnI from AS-1 have a similarity to SpnI from *S. marcescens* SS-1 (Horng et al. 2002), SprI from *S. proteamaculans* B5a (Christensen et al. 2003), SplI from *S. plymuthica* RVH1 (Van Houdt, Givskov, et al. 2007), and SplI from *S. plymuthica* G3 (Liu et al. 2011). It has reported that these LuxI homologs similarly produce 3-oxo-C6-HSL as a major product. Although *luxI* homologs in 24 complete genome sequences of *S. marcescens* were allocated on the chromosome, the only *spnI* gene in AS-1 was located on the plasmid pSERAS01. The putative transposase and *pinR* gene were located in the upstream region of the *spnIR* genes in the pSERAS01 plasmid (fig. 4). These two genes are also located upstream of the *spnIR* gene in the SS-1 genome, showing high homology with those from AS-1 (fig. 4). It has been considered that the *spnIR* quorum-sensing system was carried by the transposon in SS-1 (Wei et al. 2006). Therefore, it is likely that the *spnIR* quorum-sensing system was transferred into the pSERAS01 plasmid by transposon insertions in AS-1.

The *pig* gene cluster has been identified from two *Serratia* strains, *S. marcescens* ATCC 274 and *Serratia* sp. ATCC 39006 (Harris et al. 2004). The results of the BLAST search identified that only seven strains belonging to the clade III contained the *pig* gene cluster in 35 complete genome sequences of *S. marcescens* and shared high identity (over 98%) to the *pig* gene cluster of *S. marcescens* ATCC 274 (fig. 1). The *pig* gene cluster was seated between gene homologs involved in MerR family transcriptional regulator (*cueR*) and copper exporting ATPase (*copA*), but completely disappeared between *cueR* and *copA* in the genome of Pig-negative strains (fig. 3*c*). It has been reported that prodigiosin production was controlled by AHL-mediated quorum sensing in *S. marcescens* SS-1 (Horng et al. 2002), and AS-1 (Morohoshi et al. 2007). However, among 35 complete genomes of *S. marcescens*, only two strains, AS-1 and N4-5, contained both the *luxI* homolog and *pig* gene cluster in their genome (fig. 1). In addition, the five *Serratia* strains, which are 332, CAV1492, CAV1761, Db11, and UMH9, did not contain either the *luxI* homolog or the *pig* gene cluster in their genome (fig. 1). The AHL receptor SpnR acts as a negative regulator to produce prodigiosin in both AS-1 and SS-1 (Horng et al. 2002; Tao et al. 2008). The presence of SpnR is not essential for prodigiosin production, because prodigiosin is constitutively produced in the SpnR-negative mutant of *S. marcescens* (Wei et al. 2006; Tao et al. 2008). It is therefore assumed that prodigiosin production and its regulation by quorum sensing are not essential for the life cycle of *S. marcescens*.

## Supplementary Material

Supplementary data are available at *Genome Biology and Evolution* online.

## Supplementary Material

Supplementary Data

## Notes

**Data deposition:** The complete genome sequence of *Serratia marcescens* AS-1 has been deposited in the DDBJ/ENA/GenBank databases under accession numbers AP019009 (chromosome) and AP019010 (plasmid pSERAS01).
