# Supplementary material for: Distribution and Genetic Diversity of Genes Involved in Quorum Sensing and Prodigiosin Biosynthesis in the Complete Genome Sequences of Serratia marcescens
Source: Genome Biol Evol. 2019 Mar 6;11(3):931–6. doi: 10.1093/gbe/evz046 (PMC6433178; doi:10.1093/gbe/evz046)
Supplement: Supplementary Data [file evz046_supp.docx]

FIG. S1. Graphical representation of the complete genome of *S. marcescens* AS-1. The complete genome of AS-1 is composed of a single chromosome (a) and a single plasmid pSERAS01 (b) which consists of 5,071,908 and 104,121 bp, respectively. Concentric rings, numbered from outer to inner rings, represent the following: CDS (light blue), rRNA genes (orange), tRNA genes (pink), GC content as a peak to valley profile (black), GC-skew graph (purple and green). These graphical maps were generated using the CGView server.

| Strains | Nucleotide size (bp) | | | | | | Accession  numbers | Source |
| --- | --- | --- | --- | --- | --- | --- | --- | --- |
|  | chromosome | plasmid1 | plasmid2 | plasmid3 | plasmid4 | plasmid5 |  |  |
| AS-1 | 5071908 | 104121 | － | － | － | － | AP019009-10 | soil |
| 332 | 5059456 | － | － | － | － | － | CP021164 | clinical isolate |
| AR_0027 | 5472946 | 22569 | － | － | － | － | CP026702-3 | clinical isolate |
| AR_0091 | 5309542 | － | － | － | － | － | CP027533 | clinical isolate |
| AR_0099 | 5284831 | － | － | － | － | － | CP027539 | clinical isolate |
| AR_0121 | 5140918 | － | － | － | － | － | CP028949 | clinical isolate |
| AR_0122 | 5140912 | － | － | － | － | － | CP029746 | clinical isolate |
| AR_0123 | 5140937 | － | － | － | － | － | CP028948 | clinical isolate |
| AR_0124 | 5180277 | － | － | － | － | － | CP028946 | clinical isolate |
| AR_0130 | 5138817 | － | － | － | － | － | CP028947 | clinical isolate |
| AR_0131 | 5140937 | － | － | － | － | － | CP029715 | clinical isolate |
| B3R3 | 5471721 | 123171 | － | － | － | － | CP013046-7 | *Zea mays* |
| CAV1492 | 5477084 | 199444 | 73100 | 69158 | 6393 | 3223 | CP011637-42 | clinical isolate |
| CAV1761 | 5540160 | 204825 | 73100 | 69158 | 6393 | 3223 | CP029444-9 | clinical isolate |
| Db11 | 5113802 | － | － | － | － | － | HG326223 | insect |
| FDAARGOS_65 | 5248423 | － | － | － | － | － | CP026050 | clinical isolate |
| N4-5 | 5074473 | 11089 | － | － | － | － | CP031315-6 | soil |
| RSC-14 | 5127030 | － | － | － | － | － | CP012639 | *Solanum nigrum* |
| SGAir0764 | 5142714 | 76484 | － | － | － | － | CP027300-1 | air |
| SM39 | 5225577 | 58929 | 41517 | － | － | － | AP013063-5 | clinical isolate |
| SMB2099 | 5123091 | － | － | － | － | － | HG738868 | clinical isolate |
| SmUNAM836 | 5207023 | 26346 | － | － | － | － | CP012685-6 | clinical isolate |
| U36365 | 5125866 | － | － | － | － | － | CP016032 | clinical isolate |
| UMH1 | 5056149 | 73532 | － | － | － | － | CP018915-6 | clinical isolate |
| UMH2 | 5308626 | － | － | － | － | － | CP018924 | clinical isolate |
| UMH3 | 5300955 | － | － | － | － | － | CP018925 | clinical isolate |
| UMH5 | 5357156 | 100699 | － | － | － | － | CP018917-8 | clinical isolate |
| UMH6 | 5192910 | － | － | － | － | － | CP018926 | clinical isolate |
| UMH7 | 5182841 | 111810 | 47264 | 21738 | － | － | CP018919-22 | clinical isolate |
| UMH8 | 5155137 | － | － | － | － | － | CP018927 | clinical isolate |
| UMH9 | 5024591 | － | － | － | － | － | CP018923 | clinical isolate |
| UMH10 | 5221713 | － | － | － | － | － | CP018928 | clinical isolate |
| UMH11 | 5221717 | － | － | － | － | － | CP018929 | clinical isolate |
| UMH12 | 5196805 | － | － | － | － | － | CP018930 | clinical isolate |
| WW4 | 5241455 | 3248 | － | － | － | － | CP003959-60 | paper machine |

Table S1. Summary of the complete genome information of *Serratia marcescens* strains AS-1 and 34 in DDBJ/ENA/GenBank databases
